# Diagnostic performance of Plasma *p*‐tau217 and Aβ42/40 Biomarkers in an Outpatient Memory Clinic

**DOI:** 10.1002/alz70856_104860

**Published:** 2026-01-07

**Authors:** Yoav D Piura, Daniel Figdore, Alicia Algeciras‐Schimnich, Joshua A Bornhorst, Christian Lachner, Neill R. Graff‐Radford, Gregory S Day

**Affiliations:** ^1^ Mayo Clinic in Florida, Jacksonville, FL, USA; ^2^ Mayo Clinic, Rochester, MN, USA

## Abstract

**Background:**

Plasma *p*‐tau217 represents a promising alternative to PET‐ and CSF‐based biomarkers of AD in well‐characterized research cohorts. However, the performance of this accessible biomarker in outpatient clinics comprised of patients with multiple causes of cognitive impairment has yet to be established.

**Method:**

Plasma Aβ42/40 and *p*‐tau217 concentrations were measured using Fujirebio Lumipulse assays in patients evaluated within a subspecialty memory clinic at Mayo Clinic in Florida. Syndromic and etiologic diagnoses were established by consensus, integrating clinical data and findings from MRI (*n* = 484), FDG‐PET (*n* = 117), and CSF AD biomarkers (*n* = 440). Plasma biomarker performance was evaluated using established diagnostic cutpoints to identify patients with “positive/elevated”, “intermediate”, or “negligible” levels of cerebral amyloid pathology on amyloid‐PET.

**Result:**

Plasma AD biomarkers were measured in 509 patients (mean 68.6±9.3 years; 47% female; 91.3% non‐Hispanic White), including patients with typical amnestic AD (*n* = 237, 46.6%), non‐amnestic presentations of AD (*n* = 48, 9.4%), and non‐AD causes of cognitive concerns (*n* = 224, 44.0%). “Intermediate” plasma *p*‐tau217 and *p*‐tau217/Aβ42 concentrations were reported in <15% of patients (43% of patients with Aβ42/40), implying the need for further testing to inform the likelihood of AD neuropathologic change in these patients. After excluding these patients, plasma *p*‐tau217 reliably distinguished patients with symptomatic AD with 95% sensitivity and 82% specificity. Integration of Aβ42 measures (ptau217/Aβ42) incrementally improved specificity (86%). Diagnostic performance was unaffected by age (> vs < 65‐years). Plasma *p*‐tau217 (AUC: 0.94; 95%CI: 0.91‐0.96) and *p*‐tau217/Aβ42 performance (AUC: 0.96; 95%CI: 0.94‐0.98) were closely aligned with results of established CSF AD biomarkers (*p*‐tau181/Aβ42), outperforming Aβ42/40 (AUC: 0.78; 95%CI: 0.74‐0.83). Reduced kidney function was associated with elevated plasma *p*‐tau217 and *p*‐tau217/Aβ42 concentrations in patients without AD (referencing CSF AD biomarkers).

**Conclusion:**

Concentrations of plasma *p*‐tau217, but not Aβ42/40, strongly associated with clinical diagnoses of symptomatic AD and CSF AD biomarker results. Integration of Aβ42 measures yielded marginal improvements in performance, with increased complexity and cost. These findings support the use of plasma *p*‐tau217 in heterogeneous clinical cohorts, including patients with multiple causes of cognitive impairment. Caution is advised when interpreting *p*‐tau217 concentrations in patients with decreased kidney function, due to potential elevations in plasma biomarker concentrations.

